# Pyrolysis characteristics and kinetics of lignin derived from enzymatic hydrolysis residue of bamboo pretreated with white-rot fungus

**DOI:** 10.1186/s13068-016-0489-y

**Published:** 2016-03-31

**Authors:** Keliang Yan, Fang Liu, Qing Chen, Ming Ke, Xin Huang, Weiyao Hu, Bo Zhou, Xiaoyu Zhang, Hongbo Yu

**Affiliations:** Technology Center of China Tobacco, Yunnan Industrial Co., Ltd, Kunming, 650000 People’s Republic of China; College of Life Science and Technology, Huazhong University of Science and Technology, Wuhan, 430074 People’s Republic of China

**Keywords:** Pyrolysis, Bamboo, Biological pretreatment, EHRL, Thermogravimetric analysis, Py–GC/MS, Kinetics

## Abstract

**Background:**

The lignocellulose biorefinery based on the sugar platform usually focuses on polysaccharide bioconversion, while lignin is only burned for energy recovery. Pyrolysis can provide a novel route for the efficient utilization of residual lignin obtained from the enzymatic hydrolysis of lignocellulose. The pyrolysis characteristics of residual lignin are usually significantly affected by the pretreatment process because of structural alteration of lignin during pretreatment. In recent years, biological pretreatment using white-rot fungi has attracted extensive attention, but there are only few reports on thermal conversion of lignin derived from enzymatic hydrolysis residue (EHRL) of the bio-pretreated lignocellulose. Therefore, the study investigated the pyrolysis characteristics and kinetics of EHRL obtained from bamboo pretreated with *Echinodontium taxodii* in order to evaluate the potential of thermal conversion processes of EHRL.

**Results:**

Fourier transform infrared spectroscopy spectra showed that EHRL of bamboo treated with *E. taxodii* had the typical lignin structure, but aromatic skeletal carbon and side chain of lignin were partially altered by the fungus. Thermogravimetric analysis indicated that EHRL pyrolysis at different heating rates could be divided into two depolymerization stages and covered a wide temperature range from 500 to 900 K. The thermal decomposition reaction can be well described by two third-order reactions. The kinetics study indicated that the EHRL of bamboo treated with white-rot fungus had lower apparent activation energies, lower peak temperatures of pyrolysis reaction, and higher char residue than the EHRL of raw bamboo. Pyrolysis–gas chromatography–mass spectrometry (Py–GC/MS) was applied to characterize the fast pyrolysis products of EHRL at 600 ℃. The ratios of guaiacyl-type to syringyl-type derivatives yield (G/S) and guaiacyl-type to p-hydroxy-phenylpropane-type derivatives yield (G/H) for the treated sample were increased by 33.18 and 25.30 % in comparison with the raw bamboo, respectively.

**Conclusions:**

The structural alterations of lignin during pretreatment can decrease the thermal stability of EHRL from the bio-treated bamboo and concentrate the guaiacyl-type derivatives in the fast pyrolysis products. Thus, the pyrolysis can be a promising route for effective utilization of the enzymatic hydrolysis residue from bio-pretreated lignocellulose.

## Background

Lignocellulose biorefinery based on sugar platform can deliver a sustainable approach to industrial-scale production of renewable biofuels and chemicals [1, 2]. Traditional industrial processes for sugar platform biorefinery usually focus on the bioconversion of polysaccharides in the lignocellulosic biomass, including pretreatment, enzymatic saccharification, and fermentation to ethanol or other bio-chemicals, while lignin in the enzymatic hydrolysis residue is only burned to generate the power required in the production [3]. Lignin composed of aromatic compounds is the second most abundant bio-macromolecule after cellulose in nature, and it has a great potential for the conversion into liquid transportation fuel and value-added chemicals [4]. Development and scale-up of biorefinery industry might yield substantial solid residue with high lignin content [5, 6]. So it is necessary to promote the integration of biorefinery processes and to improve the cost competitiveness of the industry by developing novel routes for effective utilization of lignin [4, 7].

The recent trend of biofuel and chemical recovery from lignin has led to a growing interest in the area of fast pyrolysis [8, 9]. Fast pyrolysis provided a fast heating process of feedstock at 450–650 °C under the anoxic condition. Lignin can be transformed into the bio-oil consisting of valuable aromatic hydrocarbons and the solid char by the fast pyrolysis [10–12]. Currently, pyrolysis characteristics of lignin derived from a wide variety of biomass feedstocks have been investigated using thermogravimetric analysis (TGA), pyrolysis–gas chromatography/mass spectrometry (Py–GC/MS), or Fourier transform infrared spectrometry (FTIR) technologies [13, 14]. The pyrolysis of lignin derived from enzymatic hydrolysis residue may be distinctly different from that of natural lignin because the chemical and physical structure of lignin can be altered significantly by the pretreatment process which is a conventional method to make the cellulose more accessible to enzymatic hydrolysis during lignocellulose bioconversion [15]. The alteration is usually depending on the type and condition of pretreatment process [16]. In the past 10 years, biological pretreatment technology using white-rot fungi has attracted extensive attention because it was more environmentally benign and had less energy consumption [17, 18]. The fungal pretreatment can overcome barriers of biomass structure and facilitate subsequent enzymatic hydrolysis by selective delignification [19]. Large quantities of researches on developing white-rot fungal pretreatment processes have also been reported, including screening fungal strains for specific biomass, optimizing biopretreatment condition, and combining chemical processes with biopretreatment [20–22]. After fungal delignification, about 40–60 % of bio-altered lignin is still left in the biomass. Because the composition and yield of pyrolysis product are greatly affected by the chemical structure and source of lignin, the bio-modification might change thermal decomposition behavior of lignin in hydrolysis residues and further facilitated the lignin recovery and thermal conversion [13]. However, there are few reports on thermal conversion of lignin derived from enzymatic hydrolysis residue after fungal pretreatment.

Moso bamboo (*Phyllostachys pubescens*) is one of the most important fast-growing tree species for industrial purposes in China. It covers more than 3 million hectares of area and the annual yield is approximately 18 million tons [23]. Recent researches indicated that bamboo is a promising candidate substrate for fuel ethanol production [24, 25]. Our previous study has also demonstrated that white-rot fungus *Echinodontium taxodii* can be used in the biological pretreatment to improve enzymatic conversion of moso bamboo to a great extent [22]. A stream of bio-modified lignin with supernumerary conversion potential for biofuels will be introduced into the process when fungal pretreatment enters into the lignocellulose biorefinery. Although the thermal behaviors of bio-pretreated lignocellulose have been investigated widely, study on thermal conversion of bio-modified lignin still remains to be explored [26]. Therefore, the present study explored the pyrolysis behavior of lignin derived from enzymatic hydrolysis residue (EHRL) of bamboo after fungal pretreatment to evaluate thermal conversion potential of bio-modified lignin to chemicals. The pyrolysis kinetics of the bio-modified lignin was investigated through TGA. Moreover, PY–GC/MS was applied to characterize the fast pyrolysis products of the lignin.

## Results and discussion

### Structural characterization of lignin derived from hydrolysis residue

**Fig. 1:**
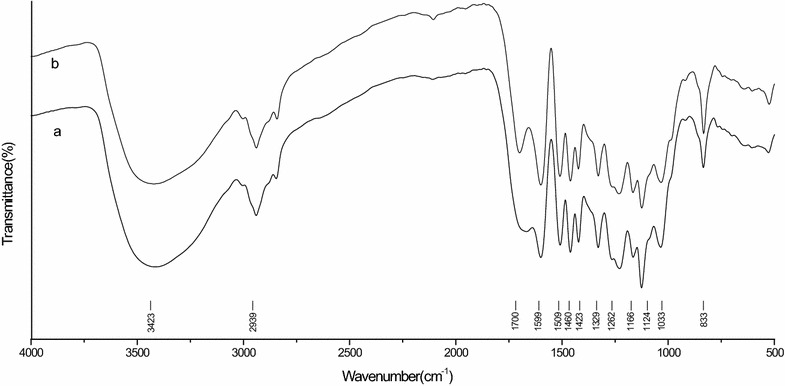
Fourier transform infrared spectroscopy analysis of EHRL. *a* EHRL of bamboo pretreated with *E. taxodii* and *b* EHRL of untreated bamboo

Fourier transform infrared spectroscopy (FTIR) analysis was used in the characterization of EHRL of bamboo pretreated with *E. taxodii*. Figure 1 shows FTIR spectra of EHRL of treated bamboo, and the absorption peaks were assigned according to references [27–29]. The structure of EHRL of untreated bamboo, natural lignin structure, was also characterized by FTIR as a control. The absorption at 3423 cm^−1^ is attributed to O–H stretching vibration in aromatic and aliphatic OH groups, and the absorption bands at 2939 cm^−1^ are assigned to C–H stretching vibration of saturated CH_2_ in side chain of lignin. The peaks between 800 and 1800 cm^−1^ represent typical lignin structure. The band at 1700 cm^−1^ indicated the presence of unconjugated carbonyl groups in ketone or aldehyde groups. The aromatic skeleton vibration in the lignin fractions occurs at 1599, 1509, 1460, and 1423 cm^−1^. The bands at 1329 and 1124 cm^−1^ indicated the existence of syringyl (S) unit of lignin, and that at 1262 cm^−1^ represented guaiacyl (G) unit of lignin. The adsorption at 1166 cm^−1^ corresponds to conjugated ester-based C=O stretching vibration that represents typical characteristics of HGS-lignin. The adsorption at 833 cm^−1^ is attributed to C–H out of plane of C2 and C6 in S unit and in all position of p-hydroxy phenylpropane (H) unit. The FTIR spectra showed that EHRL of bamboo treated with *E. taxodii* still exhibited the typical lignin structure, but the peak intensities were different from those of untreated bamboo. For example, the decreasing intensities at 1599 and 1509 cm^−1^ were observed when comparing with control, which indicated that *E. taxodii* can destroy the aromatic skeletal carbon of lignin during pretreatment. In addition, the fungal pretreatment also led to a great decrease in the relative intensity at 1700 cm^−1^, which may be attributed to the lignin side chain oxidation. This phenomenon has also been observed in the study of laccase-mediator system degrading jute lignin [30, 31].

**Fig. 2:**
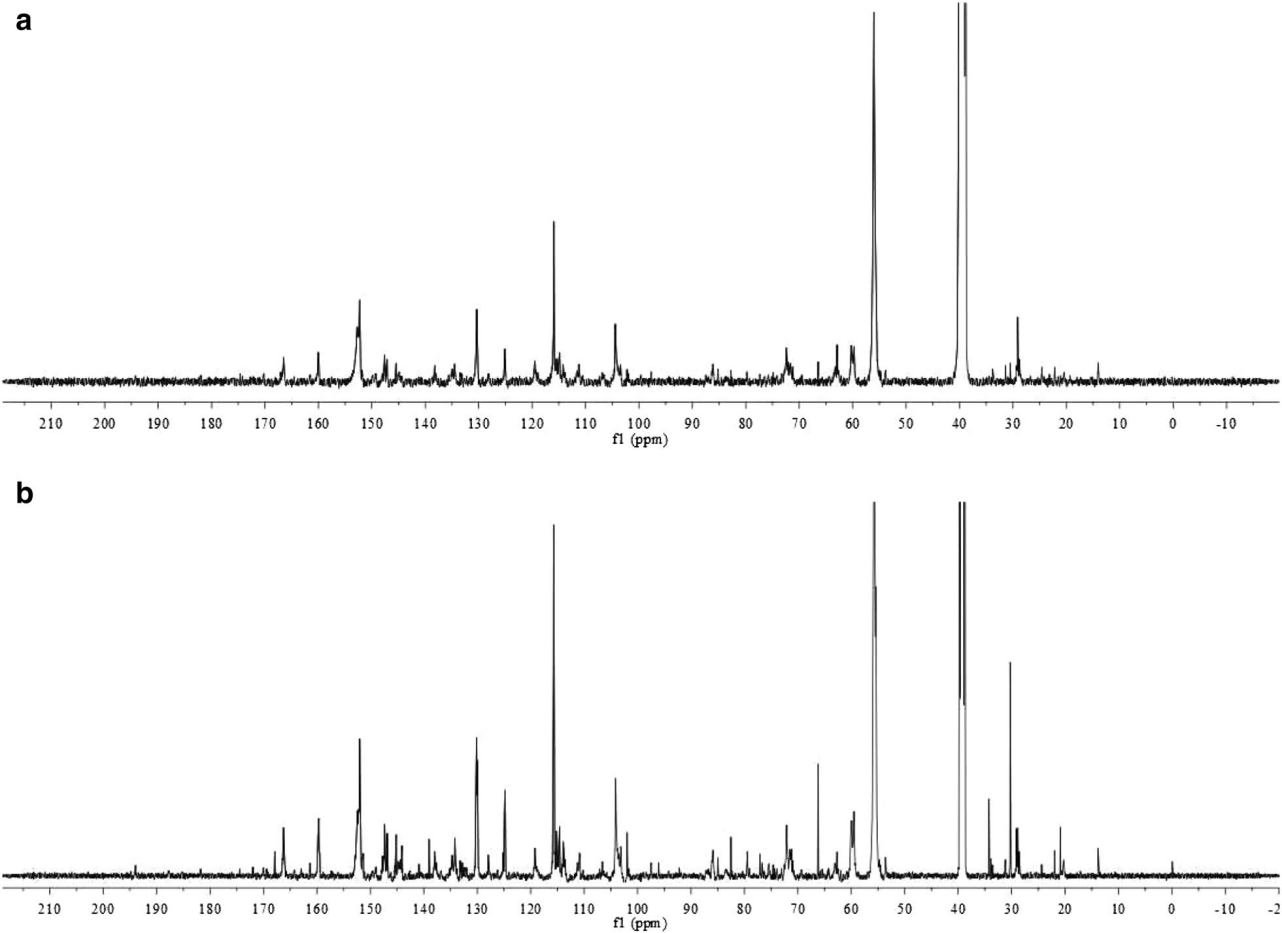
^13^C NMR spectra of EHRL. **a** EHRL of bamboo pretreated with *E. taxodii* and **b** EHRL of untreated bamboo

To further clarify the structure of EHRL, the ^13^C NMR analysis was conducted (Fig. 2). Most of the observed signals have been previously assigned in wood and bamboo lignin spectra [32, 33]. The integral of 160–102 ppm region can be used as the reference, assuming that it includes six aromatic carbons, so the integral values of all moieties can be expressed per one aromatic ring (Ar) [32]. In accordance with FTIR analysis, ^13^C NMR spectrum showed that EHRL of the treated bamboo has the typical signals of lignin. The spectra of EHRL showed seven typical strong signals derived from C8 in *p*-coumaric ester, C8, C3/C5, C1, C2/C6, C7, C4, and C9 at 115.0, 116.0, 125.1, 130.3, 144.7, 160.0, and 166.5 ppm, respectively [34]. In addition, the signals of S, G, and H were observed in the spectrum of EHRLs from both treated and untreated samples according to references. These signals were at 104.4, 106.9, 111.1, 114.8, 119.8, 128.1, 132.7–133.4, 134.5–134.7, 138.2, 145.1–145.5, 148.0–148.4, 149.7, and 152.5 ppm [33]. However, the lignin unit contents of treated bamboo were different from those of untreated sample. G/S ratio can be calculated based on the number of carbons per Ar in the syringyl (C-2/C-6) and guaiacyl (C-2) unit [32]. The result showed that G/S value of EHRL was increased from 0.42 to 0.49 after biological pretreatment, which suggested that *E. taxodii* could preferentially degrade S-type lignin unit. Furthermore, the spectrum of EHRL showed the signals of β-O-4′ linkages at 59.6, 60.7, 71.4, 72.2, 85.1, and 86.0 ppm [32]. The signal of –OCH_3_ groups was also observed at 55.9 ppm. It can be found that the amount of β-O-4′ structure in the treated EHRL was decreased from 0.72/Ar to 0.68/Ar, which confirmed the lignin depolymerization that may facilitate the pyrolysis reaction.

### Thermal decomposition characteristics of EHRL

**Fig. 3:**
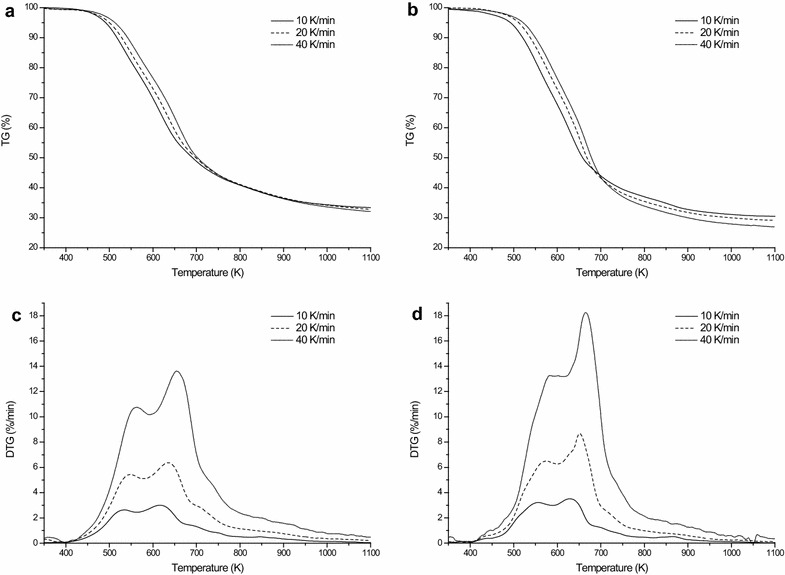
Thermogravimetric analysis of EHRL under the heating rates of 10, 20 and 40 K/min. **a**, **b** TG *curves* of EHRL from the treated and untreated bamboo, respectively; **c**, **d** DTG *curves* of EHRL from the treated and untreated bamboo, respectively

Figure 3 shows the thermogravimetric (TG) and differential thermogravimetric (DTG) curves of EHRL under the heating rates of 10, 20, and 40 K/min. The thermal decomposition of lignin covered a wide temperature range from 450 to 1100 K because it has various structures with different thermal stability [14]. The major mass loss of treated and untreated bamboo pyrolysis all occurred between 500 and 900 K at which temperature EHRL can be converted to volatile products with low molecular weight and other repolymerization products [14]. The TG curves tend to be flat when the temperature was over about 900 K at which the cracking and repolymerization of volatile products led to the formation of 25–35 wt % chars. Usually, the coniferous lignin with a dominant amount of guaiacyl unit produces more solid chars than the deciduous lignin with the similar amounts of syringyl and guaiacyl units [13]. The EHRL of treated bamboo had higher char residue at the final temperature than that of untreated bamboo, which could be attributed to the change of lignin unit content in EHRL during the fungal pretreatment.

Obviously, two overlapping peaks at the major pyrolysis stage can be observed in the DTG curves of EHRL. The main peak appeared at 616–664 K and the shoulder peak appeared at 535–581 K (Table 1), which indicated that the thermal depolymerization of EHRL can be divided into two stages. The weaker bonds, such as *β*–*γ* carbon bonds in side chain and –OH linked to *β* or *γ* carbon, were cleaved at the lower temperature stage, while the cleavage of the aryl ether linkages and C–C linkages in lignin monomeric unit occurred at the higher temperature stage [13, 14]. As the heating rate increases, the peak temperatures and the corresponding thermal degradation rates escalated. Interestingly, the peak temperatures of pyrolysis of treated sample were always lower than those of untreated sample at a given constant heating rate. Some lignin samples isolated by chemical method like Klason lignin (obtained by acid hydrolysis) are more resistant to pyrolysis compared to the lignin derived by enzymatic hydrolysis. Our previous study showed that the TGA under the heating rates of 10 K/min resulted in the formation of about 50 wt% chars for the acid detergent lignin preparation (ADL) which was obtained from moso bamboo by hydrolysis using 72 % H_2_SO_4_ and consists mainly of Klason lignin [26]. The thermal decomposition temperature for 10 % mass loss for ADL was about 550 K, while that for EHRL in this study was about 525 K. Pandey and Kim [14] also reported that Klason lignin has very high decomposition temperature and reached 623 K for 10 % mass loss. This is because enzymatic hydrolysis undergoes less structural change than acid hydrolysis which leads to more condensed structure of lignin. Furthermore, the decomposition temperature of the treated EHRL was only 510 K, which could be the result of the lignin bio-depolymerization. The results suggested that the bio-modification during the fungal pretreatment might diminish the temperature demand of lignin pyrolysis reaction.

**Table 1:** Pyrolysis kinetic parameters of lignin derived from enzymatic hydrolysis residue at different heating rates

EHRL	*β* (K/min)	Char (%)	First stage			Second stage		
			*E* (kJ/mol)	*A* (S^−1^)	*T*_peak_ (K)	*E* (kJ/mol)	*A* (S^−1^)	*T*_peak_ (K)
Fungal treatment	10	33.04	50.5	1.82 × 10^2^	535	57.2	7.51 × 10^2^	616
	20	32.18	51.2	3.17 × 10^2^	546	61.3	2.62 × 10^3^	634
	40	31.64	51.5	4.84 × 10^2^	558	69.7	2.11 × 10^4^	655
Non-treatment	10	30.18	50.6	1.68 × 10^2^	556	74.7	3.15 × 10^4^	630
	20	28.93	55.6	6.94 × 10^2^	573	90.5	9.80 × 10^5^	652
	40	26.66	56.7	1.28 × 10^3^	581	97.9	5.20 × 10^6^	664

### Kinetics analysis of EHRL pyrolysis

A kinetic analysis of EHRL pyrolysis was conducted in this study in order to understand further the thermal decomposition behavior of the EHRL from the bio-treated bamboo. In the gas–solid reaction, the degree of conversion *α* is usually expressed in accordance with the following equation:

1 $$\frac{{{\text{d}}\alpha }}{{{\text{d}}T}} = \frac{A}{\beta }\exp \left( { - \frac{E}{RT}} \right)\left( {1 - \alpha } \right)^{n} ,$$

where *A* is the pre-exponential factor, *E* is the apparent activation energy, *β* is the heating rate, *R* is the universal gas constant, *T* is the temperature, and *n* is the reaction order. The *α* can be also calculated by (*w*_i_−*w*_t_)/(*w*_i_−*w*_f_). Here *w*_i_, *w*_t_, and *w*_f_ are the initial weight, weight at time *t*, and final weight of the sample, respectively [26].

Upon integration, Eq. (1) gives

2 $$\int_{0}^{\alpha } {\frac{{{\text{d}}\alpha }}{{\left( {1 - \alpha } \right)^{n} }} = \frac{A}{\beta }\int_{{T_{0} }}^{T} {\exp \left( { - \frac{E}{RT}} \right){\text{d}}T} } .$$

Our previous study on acid detergent lignin pyrolysis demonstrated that the lignin pyrolysis was better described by a third-order reaction rate law, which was also demonstrated in Manyà’s study on the Kraft alkali lignin pyrolysis [26, 35]. The solution of Eq. (2) can be obtained by an asymptotic approximation method of Coats-Redfern when *n* was 3:

3 $${\text{ln}}\left[ {\frac{{\left( {1 - \alpha } \right)^{{ - 2}} - 1}}{{2T^{2} }}} \right] = \ln \left( {\frac{{AR}}{{\beta E}}\left( {1 - \frac{{2RT}}{E}} \right)} \right) - \frac{E}{R} \cdot \frac{1}{T}$$

In ln[((1−*α*)^2^−1)]/2*T*^2^] plotted against 1/*T*, the value of *E* or *A* could be calculated with the slope and intercept based on Eq. (3) since 2*RT*/*E* ≪ 1.

It was obvious that a single reaction was not sufficient to describe the two-stage pyrolysis kinetic of lignin. Therefore, the kinetic parameters of two pyrolysis stages for the low and high temperatures were calculated using Eq. (3) (Table 2). The result showed that the apparent activation energy and pre-exponential factor changed with the increasing heating rate. The activation energy of the first stage at the lower temperature was 50–57 kJ/mol, and that of the second stage at the higher temperature was 57–98 kJ/mol. The apparent activation energies of EHRL from the treated sample were always lower than those from untreated sample, which can be attributed to the fungal modification of lignin structure. The decrease in thermal stability of lignin by fungal treatment made pyrolysis easier, leading to the lower peak temperatures of EHRL from treated sample.

**Table 2:** Pyrolysis product analysis of lignin derived from enzymatic hydrolysis residue at 60 ℃

Peak	Compound	Retention time (min)	Peak area (%)	
			Non-treatment	Fungal treatment
1	Acetic acid	2.413	1.75 ± 0.16	2.12 ± 0.49
2^H^	Toluene	4.844	0.02 ± 0.03	0.28 ± 0.14
3	Furfural	6.492	0.15 ± 0.04	0.42 ± 0.08
4^H^	Phenol	11.269	0.98 ± 0.06	1.44 ± 0.17
5^H^	2-Methylphenol	13.573	0.37 ± 0.03	0.42 ± 0.01
6^H^	4-Methylphenol	14.228	1.71 ± 0.03	1.45 ± 0.27
7^G^	Guaiacol	14.630	1.48 ± 0.16	1.96 ± 0.47
8^H^	2,6-Dimethylphenol	15.137	0.13 ± 0.04	0.18 ± 0.06
9^H^	2,4-Dimethylphenol	16.405	0.53 ± 0.12	0.54 ± 0.14
10^H^	4-Ethylphenol	16.997	1.82 ± 0.24	1.49 ± 0.22
11^G^	2-Methoxy-6-methylphenol	17.314	0.46 ± 0.13	0.38 ± 0.01
12^G^	2-Methoxy-4-methylphenol,	17.758	2.11 ± 0.11	2.05 ± 0.05
13	Catechol	18.054	1.13 ± 0.07	1.49 ± 0.10
14^H^	2,3-Dihydrobenzofuran	18.561	9.28 ± 0.72	6.23 ± 0.48
15^H^	1-Ethyl-4-methoxybenzene	19.047	0.46 ± 0.12	0.34 ± 0.17
16^H^	4-(2-Propenyl)phenol	19.512	0.24 ± 0.11	0.17 ± 0.04
17^H^	4-Propylphenol	19.618	0.41 ± 0.02	0.21 ± 0.02
18^G^	3-Methoxy-2-benzenediol	19.745	2.87 ± 0.23	3.86 ± 0.06
19^G^	4-Ethylguaiacol	20.210	1.23 ± 0.08	1.17 ± 0.16
20^H^	2-Allylphenol	20.500	0.20 ± 0.07	0.21 ± 0.05
21	4-Methylcatechol	20.590	1.24 ± 0.33	0.93 ± 0.00
22^H^	4-(2-Propenyl)phenol	21.080	0.49 ± 0.31	0.18 ± 0.03
23^G^	2-Methoxy-4-vinylphenol	21.182	2.27 ± 0.26	2.3 ± 0.38
24^G^	3-Methoxy-5-methylphenol	21.372	0.48 ± 0.21	0.57 ± 0.21
25^H^	4-(2-Propenyl)phenol,	21.922	1.16 ± 0.21	0.79 ± 0.17
26^S^	Syringol	22.239	4.04 ± 0.15	4.48 ± 0.61
27^G^	Eugenol	22.344	0.58 ± 0.01	0.36 ± 0.02
28^S^	3,4-Dimethoxyphenol	22.450	1.73 ± 0.03	1.11 ± 0.17
29^H^	4-Hydroxybenzaldehyde	22.767	0.94 ± 0.07	0.36 ± 0.11
30^S^	2,6-Dimethoxytoluene	22.852	0.52 ± 0.22	ND
31^G^	Vanillin	23.486	1.33 ± 0.15	1.08 ± 0.08
32^G^	(e)-Isoeugenol	23.655	0.73 ± 0.04	0.54 ± 0.05
33^G^	Vanillic acid	24.733	6.60 ± 0.11	5.4 ± 0.41
34^G^	Homovanillyl alcohol	24.965	0.31 ± 0.03	ND
35^G^	4′-Hydroxy-3′-methoxyacetophenone	25.621	0.90 ± 0.12	1.16 ± 0.06
36^G^	Methyl vanillate	26.276	0.27 ± 0.02	0.43 ± 0.05
37^S^	1,2,3-Trimethoxy-5-methylbenzene	26.466	1.09 ± 0.05	0.98 ± 0.26
38^G^	(4-Hydroxy-3-methoxyphenyl)acetone	26.551	0.57 ± 0.13	0.54 ± 0.03
39^S^	3′,5′-Dimethoxyacetophenone	27.206	2.28 ± 0.18	1.96 ± 0.42
40^G^	Isovanillic acid	27.375	0.22 ± 0.04	0.41 ± 0.10
41^G^	4′-Hydroxy-3′-methoxyacetophenone	27.607	ND	0.44 ± 0.01
42^S^	4-Hydroxy-3,5-dimethoxyallylbenzene	27.861	1.15 ± 0.02	0.98 ± 0.07
43^S^	4-Hydroxy-3,5-dimethoxyallylbenzene	28.643	1.45 ± 0.16	1.25 ± 0.23
44^S^	Syringaldehyde	28.812	3.52 ± 0.25	1.91 ± 0.07
45^G^	Coniferyl alcohol	28.939	0.46 ± 0.05	0.33 ± 0.07
46^S^	4-Hydroxy-3,5-dimethoxyallylbenzene	29.383	3.81 ± 0.30	2.94 ± 0.28
47^S^	Acetosyringone	29.848	3.11 ± 0.68	2.56 ± 0.16
48^H^	4-Hydroxycinnamic acid methyl ester	29.996	0.74 ± 0.14	0.45 ± 0.29
49^S^	3,5-Dimethoxy-4-hydroxyphenylacetic acid	30.313	2.05 ± 0.35	1.73 ± 0.09
50^S^	Acetosyringone	30.989	0.92 ± 0.15	1.31 ± 0.01
51^S^	(E)-3,5-Dimethoxy-4-hydroxycinnamaldehyde	32.786	0.78 ± 0.37	ND
	Total peak areas of S		26.44 ± 1.64	21.22 ± 1.74
	Total peak areas of G		22.89 ± 0.33	23.01 ± 1.57
	Total peak areas of H		19.49 ± 0.21	14.71 ± 0.47

*S* syringyl-type lignin derivatives, *G* guaiacyl-type lignin derivatives, *H* p-hydroxy phenylpropane-type lignin derivatives

### Py–GC/MS analysis

**Fig. 4:**
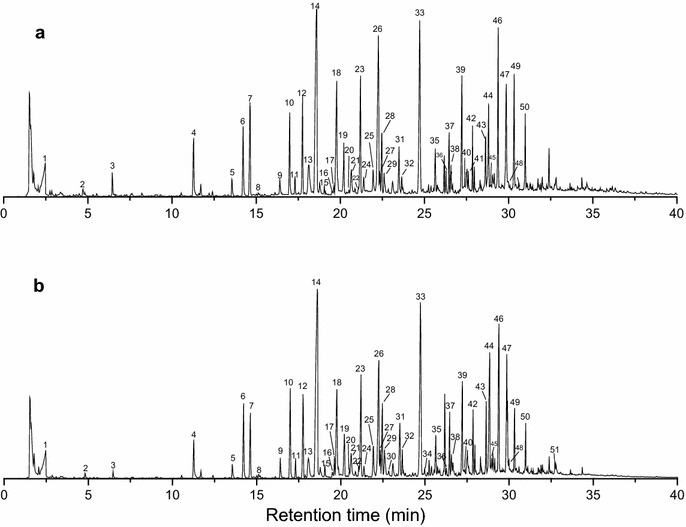
Py–GC/MS programs of EHRL at 600 °C. **a** EHRL of bamboo pretreated with *E. taxodii* and **b** EHRL of untreated bamboo

The fast pyrolysis of EHRL was conducted at 600 °C (873.15 K) that was the usual temperature for the maximum yield of monophenols from the fast lignin pyrolysis [11]. About 50 pyrolysis products were identified in the fast pyrolysis programs of EHRL from the treated and untreated samples (Fig. 4). Table 2 summarizes the major pyrolysis products according to the reported literatures and list their peak areas [8, 11, 36]. The phenolic compounds derived from lignin were predominant products because of the cleavage of the aryl ether linkages and carbon–carbon bonds in the monomeric unit of EHRL, while only two compounds derived from the carbohydrate, acetic acid, and furfural were found in the pyrolysates. The phenolic products consisted of derivatives of the lignin units (G, S, H) and catechol derivatives (catechol and 4-methylcatechol) that are the demethylated products of guaiacyl unit with fungal treatment [37].

The EHRLs of bamboo treated with and without the fungus produced similar pyrolysis products, but the relative amounts of the products were quite different. For example, 2, 3-dihydrobenzofuran (peak 14) derived from H-type lignin unit has shown the highest relative abundances in the pyrolysates of the EHRLs of treated and untreated samples [36], but it’s peak area decreased by 32.9 % after the fungal treatment. Compared with the EHRL of raw sample, the EHRL of treated sample usually produced more G-type lignin derivatives, such as guaiacol (peak 7), 3-methoxy-2-benzenediol (peak 18), 4′-hydroxy-3′-methoxyacetophenone (peaks 35, 41), and isovanillic acid (peak 40), and less S-type derivatives, such as 3,4-dimethoxyphenol (peak 28), 2,6-dimethoxytoluene (peak 30), 3′,5′-dimethoxyacetophenone (peak 39), 4-hydroxy-3,5-dimethoxyallylbenzene (peaks 42, 43, 46), syringaldehyde (peak 44), acetosyringone (peaks 47, 50), and (E)-3,5-dimethoxy-4-hydroxycinnamaldehyde (peak 51). In the pyrolysates of EHRL from untreated sample, total peak areas of G, S, and H lignin derivatives were 22.89 ± 0.33 %, 26.44 ± 1.64 %, and 19.49 ± 0.21 %, respectively. However, the total yield of G-type derivatives slightly increased when the yield of H-type and S-type derivatives from the EHRL of treated bamboo greatly reduced, and the total peak area of G, S, and H lignin derivatives was 23.01 ± 1.57 %, 21.22 ± 1.74 %, and 14.71 ± 0.47 %, respectively. Compared with the raw bamboo, the G/H and G/S ratios calculated for the treated sample were increased by 33.18 and 25.30 %, respectively. The relative content of G-type derivatives in phenolic compounds was increased by 16.65 % after fungal pretreatment. The concentration of G-type derivatives may mean demethoxylation of S units during the fungal treatment. Although G unit can also be demethoxylated and transformed into H units, it is obvious that the fungal treatment prefers to alter S and H units, which indicates that G unit was more resistant to fungal modification. This result provided a good evidence to explain why the EHRL of treated bamboo had higher char residue at the final temperature of TGA than that of untreated bamboo, because lignin with low methoxy group content (guaiacyl unit) could produce more char residue and the char yield is inversely proportional to the total amount of methoxyl groups (syringyl units) [38]. Moreover, it showed the possibility to concentrate some specific products by controlling the fungal treatment process. The fungal pretreatment is a mild and environmentally friendly process of lignocellulose biorefinery [39]. *E. taxodii* has been proved to enhance the enzymatic digestion of bamboo culms, and the sugar yield increased 8.76-fold after the pretreatment [22]. When *E. taxodii* cultivated on the natural bamboo substrate, it secretes laccase and manganese peroxidase for lignin depolymerization and modification which can facilitate the thermal conversion of lignin in enzymatic hydrolysis residue [40]. Thus, the pyrolysis will provide a promising route for the effective utilization of enzymatic hydrolysis residue when fungal pretreatment is introduced into the lignocellulose biorefinery.

## Conclusions

The thermogravimetric analysis showed that EHRL pyrolysis covered a wide temperature range from 500 to 900 K and can be divided into two depolymerization stages. Besides, the EHRL of bamboo treated with white-rot fungus has evidently different pyrolysis characteristics from natural bamboo lignin because of the structural alterations of lignin during pretreatment. Compared with the EHRL of raw bamboo, the EHRL of treated bamboo has lower apparent activation energies and peak temperatures of pyrolysis reaction, which indicated that the fungal treatment may decrease the thermal stability of lignin. Moreover, the decrease of G-type derivative content in the phenolic products of EHRL fast pyrolysis products suggested that white-rot fungi *E. taxodii* can preferentially degrade S-type lignin unit, which resulted in the increase of char residue in thermogravimetric analysis experiments. The research indicated that the fast pyrolysis can be a promising route for the conversion of bio-modified lignin to chemicals and contributes to the effective utilization of the enzymatic hydrolysis residue from bio-pretreated lignocellulose.

## Methods

### Fungal pretreatment and enzymatic hydrolysis of bamboo

White-rot fungus *Echinodontium taxodii* for pretreatment and enzymatic hydrolysis of bamboo was isolated from Shennongjia Scenic Area in Hubei of China [22]. Moso bamboo was obtained from Wuhan, China. Fungal pretreatment was carried out in 250-mL flasks containing 6 g (dry mass) of pulverized bamboo (grain diameter = 0.3–0.45 mm) and 13.5 mL of distilled water. The treated bamboo was dried (in an aerated oven at 60 °C) after incubation for 30 days at 28 °C. Untreated sample was incubated under the same conditions. Enzymatic hydrolysis of the treated and untreated samples was conducted at 2.5 % substrate concentration in 50 mM sodium acetate buffer (pH 4.8, which contained 0.01 % NaN_3_ (w/v) with cellulase 30 FPU g^−1^ substrate) at 48 °C for 3 days. Cellulase was obtained from Sigma. After hydrolysis, the hydrolysis residues were isolated by centrifugation at 10,000 rpm for 5 min and then freeze-dried for the preparation of lignin derived from enzymatic hydrolysis residue (EHRL).

### Preparation of EHRL

The enzymatic hydrolysis residues of bamboo were ball-milled by planetary ball mill for 7 days after acetone Soxhlet extraction, and further treated with cellulase (100 FPU g^−1^ substrate, pH 4.8, 50 mM acetate buffer, at 48 °C) for 3 days in order to remove the residual polysaccharide [28]. The insoluble materials obtained by centrifugation were washed twice with acidified deionized water and then hydrolyzed using an azeotrope of aqueous dioxane (dioxane/water 85:15, v/v, contained 0.01 mol/L HCl) under a nitrogen atmosphere. After centrifugation, the supernatant was neutralized with sodium bicarbonate, concentrated by vacuum rotary evaporation and added dropwise to the acidified deionized water (pH 2.0). The mixture was maintained overnight and was centrifuged. The precipitated EHRL was washed twice with deionized water and freeze-dried for thermal decomposition. The lignin content was measured according to the procedures of Klason analysis, and the lignin contents in the EHRL of raw and treated bamboo are 89.3 and 86.4 %, respectively [41].

### Structural characterization of EHRL

2 mg of EHRL sample was mixed with 70 mg of KBr and prepared in the form of pellets at 1 MPa for Fourier transform infrared spectroscopy (FTIR) analysis. FTIR spectra of EHRL were recorded with a NEXUS 670 spectrometer (Thermo Nicolet Corp., Madison, WI). ^13^C NMR spectra were recorded with a Bruker AV 400 MHz spectrometer at 25 °C in DMSO-d6. For ^13^C NMR experiments, 140 mg of lignin was dissolved in 0.5 ml DMSO-d6. The spectra were recorded in FT mode at 100.6 MHz. The inverse-gated-decoupling sequence was used with the parameters of 2 s relaxation delay, 1.4 s acquisition time, 64 K data points, 30° pulse angle, and 30.000 scans.

### Pyrolysis kinetic analysis

TG/DTA experiments were performed with sensitive thermobalance (Perkin–Elmer, Diamond) by Analytical and Testing Center, Huazhong University of Science and Technology [26]. Prior to thermogravimetric experiments, EHRL samples were grounded to small chips followed by a 0.15–0.2 mm mesh screening. Initial sample masses of 5 mg were placed in the pan of the TGA microbalance, which were enough to fill the pan because of the low density of the ground samples. Nitrogen was used as a carrier gas. Experiments were carried out on a thermobalance from 450 to 1100 K at the heating rates of 10, 20, and 40 K/min under a nitrogen flow of 100 mL/min.

### Py–GC/MS analysis

Fast pyrolysis tests were performed using a Pyroprobe 5200 analytical pyrolyzer (CDS Analytical Inc.) at 600 ℃ for 60 s. The pyrolysis volatiles were analyzed using a gas chromatograph (Agilent 6890 N) equipped with a mass spectrometer (5975CMSD) and DB-5 MS column. The chromatograph program was set as follows: 3 min isothermal at 40 °C, followed by 5 ℃/min to 150 °C and 10 ℃/min to 250 °C, and finally maintained at 250 °C for 25 min. The MS was performed under 70 eV EI conditions with the range from m/z 29 to 600. The pyrolysis products were identified based on the reported literature and by comparing the mass spectra with the NIST mass spectrum library [8, 11, 36].

## Abbreviations

EHRL: lignin derived from enzymatic hydrolysis residue
Py–GC/MS: pyrolysis–gas chromatography–mass spectrometry
*E. taxodii*: *Echinodontium taxodii*
FTIR: Fourier transform infrared spectroscopy
TG: thermogravimetric
DTG: differential thermogravimetric
TGA: thermogravimetric analysis
S: syringyl
G: guaiacyl
H: p-hydroxy phenylpropane
